# Identifying the Intents Behind Website Visits by Employing Unsupervised Machine Learning Models

**DOI:** 10.1007/s40745-024-00586-5

**Published:** 2025-01-09

**Authors:** Judah Soobramoney, Retius Chifurira, Temesgen Zewotir, Knowledge Chinhamu

**Affiliations:** 1 School of Mathematics, Statistics and Computer Science, University of KwaZulu-Natal, Durban, South Africa

**Keywords:** Cubic clustering criteria, Dbscan, Dendrogram, Google analytics tracking, Hierarchical clustering, K-means, Online website visits, Silhouette’s coefficient, Unsupervised machine learning

## Abstract

With digitisation globally on the rise, corporates are compelled to better understand the usage of their websites. In doing so, corporates will be empowered to better understand consumers, and make necessary adjustments to ultimately improve the corporate’s stance in the competitive global landscape of this modern age. However, the online website visit data has proven to be highly complex, big in data volume, and highly transactional with users expressing unique behaviours. Thus, extracting insight can be a complex problem to solve. This study aimed to employ unsupervised machine learning models to identify the intentions behind the visits on the observed website. The data studied was sourced from the Google Analytics tracking tool that was deployed on a corporate informative website. The study employed a k-means, hierarchical and dbscan unsupervised machine learning models to understand the intents behind visitors on the studied website. All three models detected five major intents that were expressed within the observed data. The intents identified were labelled as “accidentals”, “drop-offs”, “engrossed”, “get-in-touch” and “seekers”. On the observed data, all three unsupervised machine learning methods have performed well. However, in the context of the study, which investigated the intents that drove online visits, the hierarchical clustering method yielded superior results by maintaining the best balance between cluster homogeneity (stronger silhouette coefficients) and cluster size.

## 1 Introduction

In a digital world, corporate’s leverage of web analytics to optimize their offerings, image and operations [1, 2]. Web analytics entails the study of a website’s users’ online activity for business intel. Tracking tools such as Google Analytics tracking empower website owners to have sight of the volume of visitors that enter the website, the web pages they visit, the page path followed, the time spent on the website, the device type used, the device brand, the operating system, the coordinates of the device, the number of times the device has accessed the website, the entry point to the website and much more [3].

Data science techniques can be employed to make sense of the often big, and complex web visit data [4]. The art of data science represents an interdisciplinary field that employs statistical, specialized programming and domain knowledge to analyse big and complex data such as web visit data. In this study, the board of directors of engineering and engineering training corporate (TEKmation) were interested in the activity on its website. The business needed intel on what visitors were doing on its website [5, 6]. With a high volume of visitors with each entering the website for different intentions and following unique page paths, the data at face-value proved highly complex. To better understand the website usage in a manner that was reasonably ingestible, the study employed data science techniques through three unsupervised machine learning models to cluster the web traffic to better understand the underlying intentions inherent within the data. Three different unsupervised machine learning methods were studied and the intents that emerged are compared within this study.

### 1.1 Related Work

With the digital marketplace growing globally, such web analytics is becoming increasingly more important [7, 8]. Idowu and Kattukottai employed several clustering methods to segment online purchase data following a recency, frequency and monetary model. The study found that the hierarchical clustering method performed best within the study [9]. Porsche et al. [10] conducted a study to understand reading behaviour within online books. The main purpose of the study was to assess the performance of the Google Analytics tracking tool as a format suitable for advanced tracking of reading behaviour within webbooks, prescribe measurements for reading the behaviour of webbooks and present the results of a pilot study. Through the analytics conducted, the study suggested deployment procedures of web books and presented possible methods of web book performance evaluation. Furthermore, the study concluded that the Google Analytics tool was a valuable tool for tracking traffic to individual books and quantifying the traffic to the entire webbook collection on the observed data, through the use of unique custom and advanced metrics that were proposed [10].

Domazet and Simovic [11] conducted a study to measure the performance of an online informal educational institution through web analytics. The goal of the study sought to determine the best-performing acquisition channel for non-formal educational institutions and the aggregate visitor profile of this kind of educational program by means of visitor acquisition and behaviour data. The key metrics employed to assess the performance of the various acquisition channels were the conversion rate, average session duration, and bounce rate. However, visitor demographics (such as gender and age data) were supplemented on the side of the visitor-specific data. The findings of the study concluded favourably and suggested that the findings that emerged from this study could apply to other non-formal educational institutions alike [11].

Jonathan et al. [12] employed web analytics to understand church members’ activities on the “Church Cast” application that hosted online sermons. It was believed that the study would ultimately increase user knowledge and interaction. “Church Cast” was developed to avail sermons of the Gospel ministries more conveniently to their church members through the digital channels of the internet and mobile devices. It was believed that low-capacity church members with time constraints and religious restrictions in certain parts of the world have resulted in church members being unable to physically attend at their locations of worship to listen to or watch their ministers. The application, being web-based, tracked visitors usage and thereby informed the administrator on visitors’ activities on the application [12].

Semeradova and Weinlich [13] sought to examine web traffic of user-friendly websites and thereby proposed an analytical procedure based on data sourced from “Google Analytics” using the online interface that’s made available by Google. Semeradova and Weinlich [13] claim that web-user experience testing may often be very time-consuming, costly, and often biased, since the number of web-testers are often too few. Within the study conducted, a proposed analytical framework based on the web tracking data sourced from Google Analytics was presented. The framework proposed leveraged off the features and capabilities provided by the google analytics web-interface and introduced the concept of virtual pageviews as a user attention indicator. The study conducted A \B \C testing where each test group represented an e-shop design. The performance on each e-shop design were analysed to determine the leading e-shop design [13].

Kalyankar and Anute [14] sought to assess the value that web analytics yielded to e-commerce. The primary objective of study was to identify how web analytics was employed within the e-commerce sector. And furthermore, determine the manner in which e-commerce enhanced a corporate’s financials through web analytics. The study focused on target strategies and attempted to advocate for web analytics in sales and marketing. The findings of the study highlighted the importance of first having a strong financial goals set in order to make meaningful sense of web tracking data [14].

Cirlugea et al. [15] performed web analytics to assess the impact and optimization of Facebook advertisements on a small Romanian company that specialized in the manufacture of clothing in an artisanal and artistic manner. The primary objective of the study was to gauge the performances of Facebook adverts in terms of customer reach, engagements and reactions to the adverts, quantify the effectiveness of Facebook adverts towards the sale of the products, and additionally to establish a possible target audience for the brand. The outcomes of the study were expected to illustrate a Facebook advert guide for young niche businesses to grow [15].

Rosqa and Ati [16] conducted a study on an Indonesian corporate using web analytics in conjunction with qualitative data obtained from the corporate to assess the vital statistics of the corporate’s website (bounce rate, views, etc.). The main objective of the study was to quantify the statistical results of the “Elzatta” website analysis through the use of a web tracking tool. The study employed a combination of a qualitative approach through observation data collected at the corporate’s premises and secondary web analytics data sourced from the corporate’s website. The findings of the study indicated that the corporate’s visitors within the six-month analysis period totalled to 187,163 visitors. Furthermore, the volume of website visitors had shown to increase monthly with the most frequent visitor profile being females between the ages 18 and 34 years from the Indonesian towns of Jakarta, Surabaya and Bandung. Most website visitors have accessed the website through mobile devices that either entered the website through browsing for the website or following a social media link from Facebook and Instagram. Furthermore, the study recorded a high bounce rate of 51.48% [16].

Pirvu and Anghel [17] proposed a double recurrent neural network machine learning solution that reads visitors behaviour from the previous visits and predicts the behaviour in the current visit. Pirvu and Anghel [17] claim that e-commerce web applications were embedded within the day-to-day life of the authors and the local economy. However, most applications were believed to lack the adaptation to users’ needs and subsequently result in sub-optimal conversion rates and unsatisfied customers. The study presented a machine learning tool that learns the interests’ of visitors from previous sessions and predicts useful metrics for the current session. Thereby, these predicted metrics could inform applications to allow customisation and allow better recommendations. This would further allow presenting better offers of specific products, targeted notifications or the placing of targeted adverts. The proposed model was believed to accurately allow applications to better customise the website offers to a client’s needs and better predict a target base. The findings of the study indicated that the model was capable of yielding a probability score for each of the defined target classes [17].

Mariyapillai and Pratheepan [18] conducted a web analytics study on an online library web portal of the Uva Wellassa University (UWU) with primary interest in understanding the spatial distribution of library visitors. The study tracked a few webpages including the “Home Page”, “Online Public Access Catalogue” and “Institutional Repository”. The website analytics indicated that the studied website had been visited by roughly 366,756 local and global visitors during the period of study. On their studied website, most visitors entered the University’s website from the United States of America (15.82%) and followed by the Netherlands (4.78%) [18].

Stelian and Stoicu-Tivadar employed web analytics to study and quantify the level of interaction in a virtual reality (VR) medical application that was designed for educational purposes. The medical application allowed users to observe and handle skeletal human bones in a virtual environment. The web analytics indicated that the foot bones attained an interaction rate of 80% whilst other skeletal structures attained an interaction rate of 100% on the observed data [19].

### 1.2 Research Gap

Section 1.1 presented related research that attempted to gain insight from web visit data. Table 1 tabulates the key differences between the related work and work presented within this paper.

Given the recent literature presented in Table 1, this paper sought to cluster the underlying visit intentions behind the visits of the studied website. None of the discussed literature have indicated analysis that guided on how the underlying intentions of a website have been determined. Furthermore, several of the discussed related literature employed descriptive statistics to aggregate the complex web visit data—however descriptive aggregation is less insightful relative to the unsupervised machine learning problem detailed within this paper.

This paper attempted to determine the underlying intentions through the use of k-means, hierarchical and dbscan unsupervised machine learning models. Whilst these models are considered traditional or classical, these models are nonetheless very popular [20]. This paper serves as a comparative study to assess the ability of each of these traditional models to identify the underlying intents behind website visits on the studied informative website. Apart from their popularity, these models were selected since each belong to a different family of clustering methods: k-means belongs to the centroid-based or partition family, hierarchical clustering belongs to the connectivity-based family and the dbscan method belongs to the density-based family of clustering. Additionally, given the nature of web analytics, the audience and subsequent behaviour is highly influenced by many factors (such as geo-location, core business, aesthetics, user-experience, business seasonality [21]) thereby further adding to the novelty of this paper. Furthermore, none of the recent literature as presented in Sect. 1.1 provide any details of a visit intent clustering model.

## 2 Materials and Methods

This study employed three popular unsupervised machine learning algorithms that belong to different families of clustering methods. The k-means method belongs to the centroid-based or partition family of clustering, hierarchical clustering belongs to the connectivity-based family of clustering and the dbscan method belongs to the density-based family of clustering. Table 2 lists the notations denoted within Sect. 2.

### 2.1 K-means

The k-means algorithm follows an iterative process that partitions the dataset into *k* (pre-defined) unique subgroups (clusters) where each data point resides in one cluster alone. The algorithm strives to ensure that the data points within each cluster are as similar as possible (homogenous) whilst the clusters are as different as possible from each other cluster. The iterative process minimizes the sum of square distance between each data point and the cluster centroid. The cluster centroid represents the arithmetic mean of all data points within that cluster. The lower the variation within cluster datapoints, the more homogenous the cluster would be [22].

The pseudo code of the k-means algorithm follows the process below:

1. User specifies the number of clusters (*k*)
2. The algorithm determines the centroids by randomly selecting the *k* data points for the centroids without replacement.
3. Iterate until the is no further change to the centroids (the assignment of data points to clusters remains the same).
4. Calculate the sum of squared distance between the data points and all centroids to determine cluster membership.
5. Compute the cluster centroids by taking the average of all data points within each cluster.

### 2.2 Hierarchical Clustering

The hierarchical clustering method initiates by treating each observation as an individual cluster. Thereafter, the following two steps are iterated:

1. Identify the two clusters that are most similar
2. Thereafter, merge these two most similar clusters within this iteration.

**Fig. 1:**
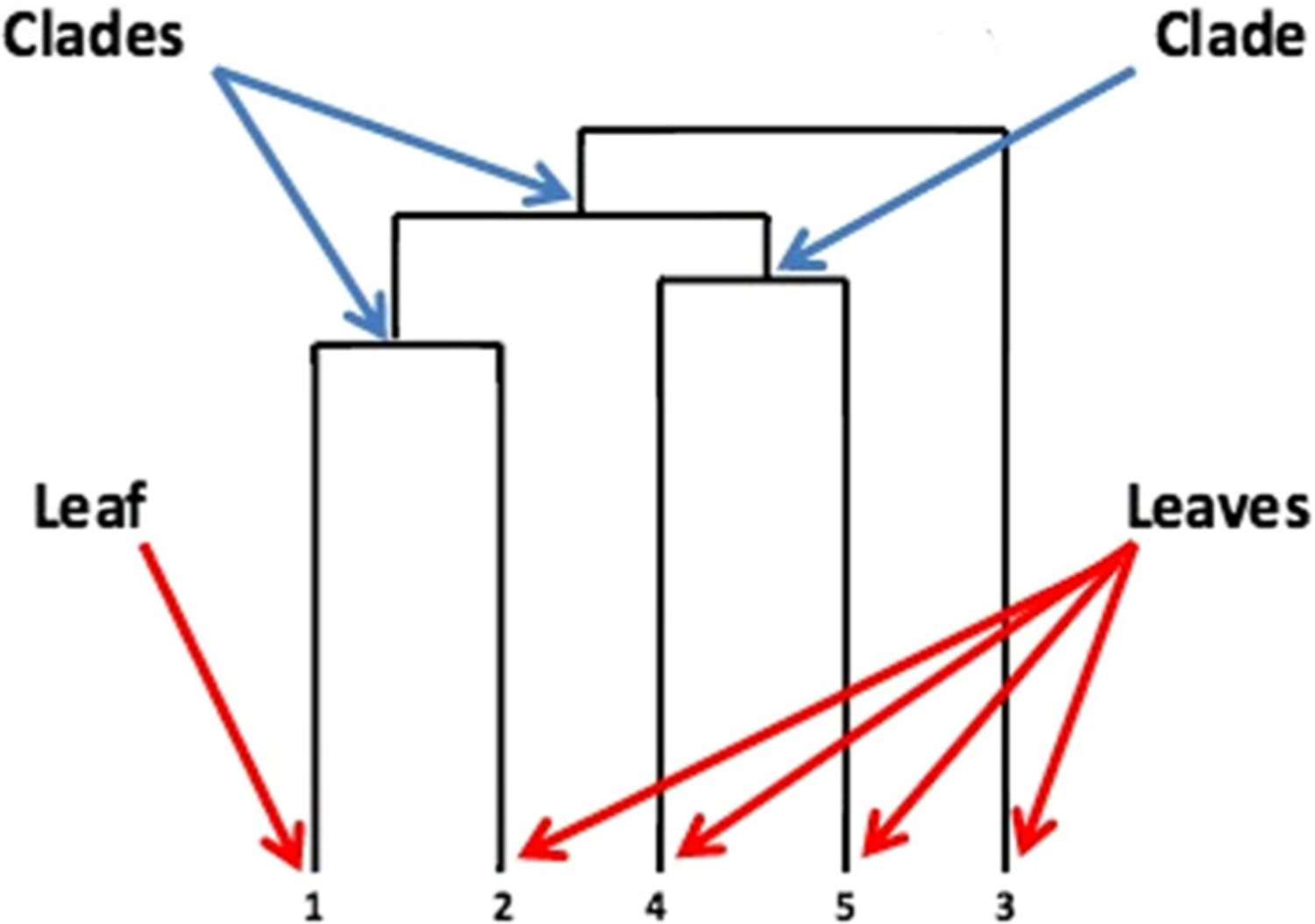
Sample dendrogram

The process continues to loop until all data-points are merged into one cluster. The dendrogram provides a visual of the hierarchical clustering process as illustrated in Fig. 1.

On a dendrogram, the clades represent the stacked branches. From bottom-up, the clades that join together first would indicate the strongest similarity [23].

### 2.3 Density-Based Spatial Clustering of Applications with Noise

The density based spatial clustering of applications with noise (dbcsan) algorithm initiates by randomly selecting a single data point (*x*) and assigns it to cluster 1. Then the algorithm assesses how many data points reside within the ϵ distance from *x*. If the count of such data points within ϵ distance is greater than or equal to the specified minimum number of points within cluster (*n*), the algorithm would then consider this data point as a core point and will assign these ϵ-neighbours to the same cluster 1. Thereafter, the algorithm examines each other member within cluster 1 and identifies their respective ϵ-neighbours. If any cluster 1 members have n or more ϵ-neighbours, these data points will be added to cluster 1. The process will continue growing cluster 1 until there are no more data points to add in (within ϵ distance and greater than or equal to the minimum number of data points). The dbscan algorithm would then randomly pick another point from the dataset not belonging to any cluster and repeat the process. Data points that did not get assigned to any cluster will be labelled as outliers [24].

Upon completion, each data point can be classified as one of the three:

- Core point: data point with at least the minimum number of neighbours within epsilon (ϵ) distance.
- Border point: data point with at least one core point within epsilon (ϵ) distance and less than the number of minimum neighbours within epsilon (ϵ) distance from itself.
- Noise point: data point with no core points within epsilon (ϵ) distance and thus could not be placed into a cluster.

### 2.4 Cubic Clustering Criterion

Across clustering methods, determining the number of clusters remains an important aspect of unsupervised machine learning. The cubic clustering criterion (CCC) is a commonly used metric to give a sense of the acceptable number of clusters and potentially, the optimal number of clusters on a given data set. The cubic clustering criteria quantifies the deviation of the clusters from the expected distribution if all data points followed a uniform distribution. According to the cubic clustering criteria, larger positive values imply a better solution, as it indicates a larger variance from a uniform (no clusters) distribution. The cubic clustering criteria values greater than 2 indicate good clusters. Cubic clustering criteria values between 0 and 2 indicate potential clusters but should be taken with caution. Furthermore, large negative values may indicate outliers. With the acceptable number of clusters in mind, the chosen number of clusters are often determined based on the profiling of the clusters in context of the data in real world applications [25].

### 2.5 Silhouette Coefficient

The silhouette coefficient is a commonly computed metric employed to assess the accuracy of clustering solutions. The silhouette coefficient ranges between −1 to +1 and imply the following [26]:

- Clusters with silhouette coefficients closer to +1 imply very tight observations within cluster (homogenous)
- Clusters with silhouette coefficients closer to 0 imply possible overlapping clusters
- Clusters with silhouette coefficients closer to −1 imply observations are not very similar.

## 3 Cluster Data

The underlying data represented within this study reflected web traffic data of a South African SMME (small, medium or micro enterprise) informative website. The online user tracking was conducted via the Google Analytics Tracking tool. A data-pipeline was constructed using R (a data-science programming language) to access the Google Analytics Tracking API and imported the data onto a local database at a non-aggregated level for further processing.

### 3.1 Feature Selection

The Google analytics platform tracks and reports on several aspects such as the device used to access the website, the geo-location co-ordinates of the device, time spent on the website, the pages visited, the number of times the user has ever been on the website, the page path followed, etc. For the unsupervised machine learning methods employed within this paper, numeric fields alone were utilized to segment the data. Table 3 provides a description of the features considered for the unsupervised machine learning models.

These are the typical numeric features that are available on web traffic data within an information website. For modelling purposes, this study analysed the data at a session level. A session (or visit) simply represents the group of interactions (pages viewed, duration, etc.) a user made while on the website in that particular instance. A user may have multiple sessions if the user visited the website several times. A feature selection process that identified redundancy (highly correlated features) and non-informative features (low variance features). The features “hits” and “pageviews” shared a high positive correlation, and thus “hits” was omitted from the model to reduce redundancy. The features “bounces” and “organicsearches” were omitted from the model due to them being binary. However, the bounce rate and organic search rates per cluster were assessed during the profiling stage. Features with natural relationships were also carefully selected. For instance, a flag that indicated if the visit was international or not would have a natural relationship with the Euclidean distance. Furthermore, pages which had minimal visits thus lacked material variation were omitted from the feature set that fed into the unsupervised machine learning models.

### 3.2 Data Cleaning

Taking the features within the consideration set for the unsupervised machine learning models, a small degree of extreme value outliers were identified and approximated through a winsorization clean to remove the influence of extreme values. Since most features were originated from the tracking tool, being system generated metrics there were no missing data points on the observed data set. Finally, the feature set was normalized to ensure a consistent scale prior to the unsupervised machine learning models.

## 4 Empirical Results

This section discusses the intents or clusters that were identified within the data by the three unsupervised machine learning methods employed.

### 4.1 Number of Clusters

**Fig. 2:**
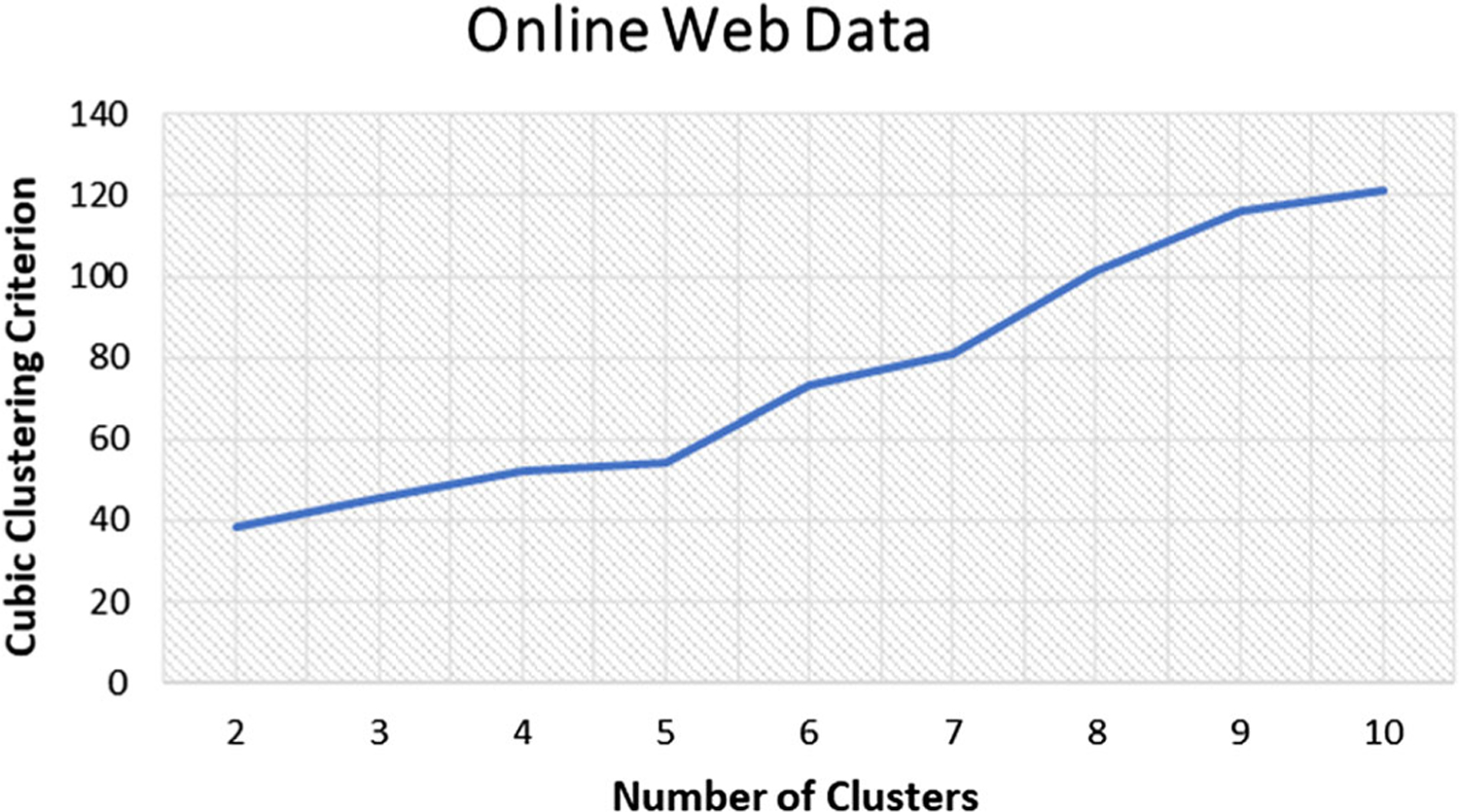
CCC values

To gauge the acceptable number of segments (intents) that prevail within the studied data, the cubic clustering criterion (CCC) metric was computed. Figure 2, illustrates the CCC metrics between a two cluster solution and a ten cluster solution.

According to the CCC measures, segmenting the data into between 2 to 10 clusters will all yield acceptable clustering solutions. However, the CCC metric also implied that the more clusters created, the better the clustering solution would be. During the study, across the several clustering methods, between 3 to 8 clustering solutions were developed. It was found that the 5 cluster solutions resulted in the most appropriate segments/intents in the context of this study across the three unsupervised machine learning methods applied. Using the clustering solutions that ranged between 3 to 8, each clustering solution was profiled by studying the cluster homogeneity across the input features (such as session duration, distance, etc.) and the corresponding cluster size to determine the optimal number of clusters. In doing so, the 5-cluster solution proved to be the most insightful across the three clustering methods.

### 4.2 K-means

This section discusses the clusters that emerged from the k-means model. The k-means model was employed to segment the online web visit data to identify the underlying intents expressed across the visits. A range of clustering solutions were developed from a three-cluster solution to an eight cluster solution. Upon profiling the data, the five-cluster solution yielded results that best explained the online web intents. Tables 4 and 5 profile the k-means five-cluster solution against various online metrics.

Table 4 presents key online metric across the five clusters whilst Table 5 indicates the average number of times cluster members visited each web page. This thereby allowed the clusters to be further understood in terms of the similarity of visits within each cluster and the differences between the clusters themselves. According to k-means model, cluster one resembled the group of visits that intended primarily to contact the corporate. During cluster one visits, the bounce rate was low, the visits were relatively in close geographic proximity to the corporate and all visitors had primary interest in the ‘contact-us’ page. Cluster two contained visits that expressed very little interest in the website. Cluster two indicated visits that would either bounce or shortly drop off the website. Furthermore, on cluster two, visitors’ geo-location were on average the furthest away from the corporate’s co-ordinates. Whilst cluster two proved to be the largest cluster identified by the k-means model, it represented a group of visits that were of very low engagement. According to the model, cluster three represented a group of visits that were fairly engrossed with a moderate engagement with the website. Cluster four represented a group of visits that were seeking specific information and dropped off thereafter. It was evident that cluster four had particular interest in the “courses” and “short-courses” webpages of the website. This page detailed the courses that the corporate had to offer. Cluster five represented a group of visits that were very engrossed and having a high engagement with the website. On cluster five, the duration and page view metrics were relatively the highest whilst the rate of organic searching (indicating that visits were not routed to the website through a link) were at the highest. Table 6 presents the average cluster silhouette scores, with cluster one and two indicating strong homogeneity. Cluster three and five grouped visits that were very engaged on the website and thus contained a wide variety of activities expressed resulting in poorer silhouette scores. Cluster four expressed a moderate silhouette score. Given the understanding behind the lower silhouette scores on cluster three and five, the k-means model yielded satisfactory results given the real-world data.

### 4.3 Hierarchical Clustering

**Fig. 3:**
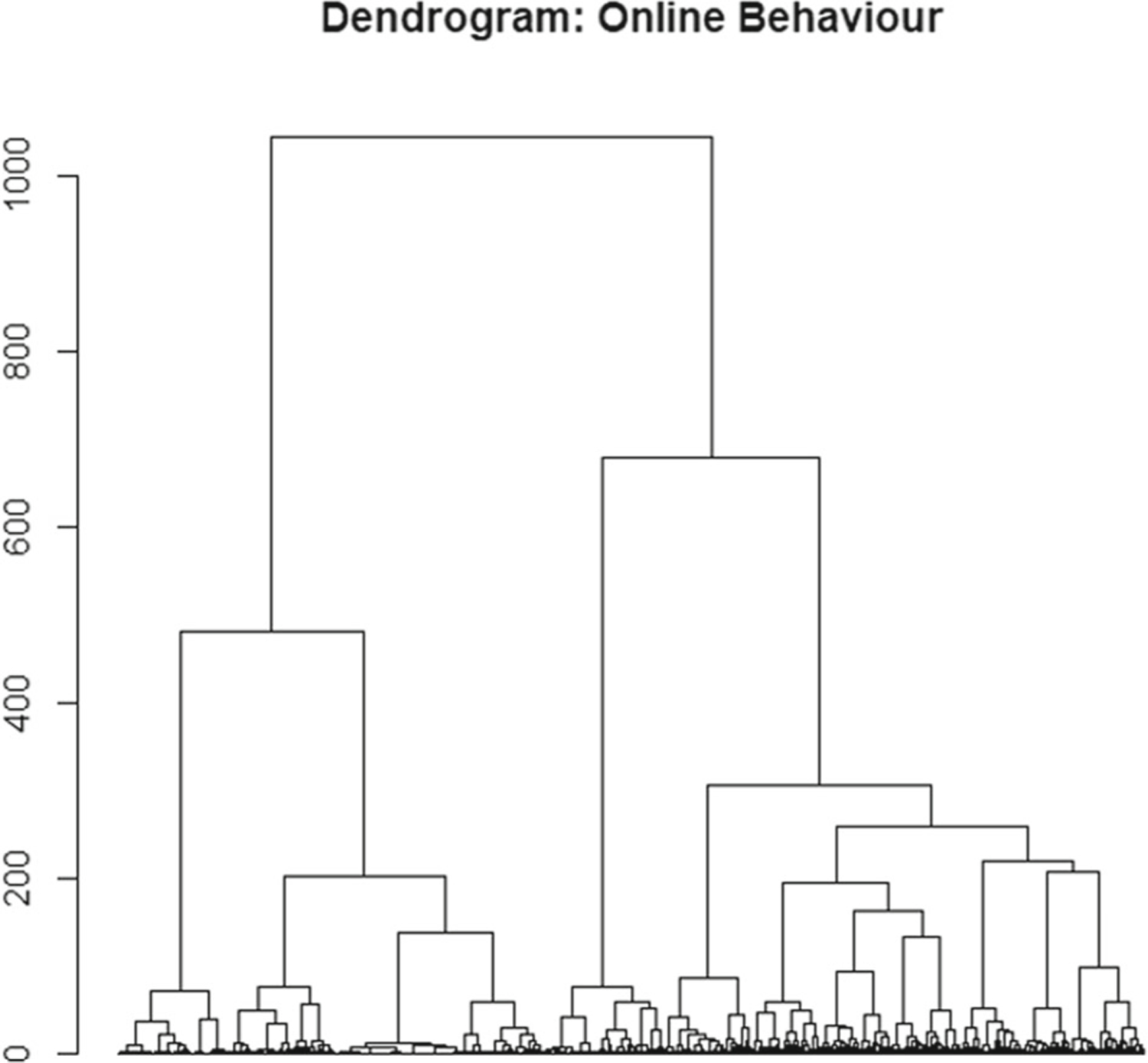
Online web visit dendrogram

The second unsupervised machine learning model employed a hierarchical clustering method. The subsequent dendrogram of the hierarchical model is presented in Fig. 3.

Upon profiling the clustering solutions, the five-cluster solution proved to be most appropriate in the context of this study. Tables 7 and 8 illustrate the cluster profiling of the hierarchical clustering model.

According to the hierarchical clustering model, cluster one represented visits that were highly engaged with the website, with high session duration, multiple page views, relatively close geographic proximity to the corporate co-ordinates and a low bounce rate. Cluster two, identified the individuals that were seeking specific information and thereafter left the website. Cluster two visits mainly went onto the “short-courses” and “course-content” pages. Cluster three, represented visits that resembled accidentally or with very little interest landing onto the website. Such visits were from the furthest geo-proximity away from the corporate’s offices (many of which were from countries outside of South Africa). Cluster three also recorded the highest bounce rate. Cluster four represented visits that showed evidence of dis-engagement due to dropping-off after spending a brief amount of time on the website. Cluster five, represented the group of visits that were primarily interested in contact information from the website and thereafter left the website. This can be inferred from the short visit duration but all visits indexing high on the “contact-us” web page. The cluster silhouette coefficients of the hierarchical clustering model are recorded in Table 9. Clusters three, four and five attained strong silhouette scores implying good cluster homogeneity. Cluster two attained a moderate silhouette score. Cluster one recorded a low silhouette score due to the nature of the visits grouped. Within cluster one, visits that were very engaged with the website and expressed their high-engagement in various ways (following unique paths and visiting pages in differing manners) and thus mathematically resulted in poor silhouette scores. Overall, the silhouette scores implied that the hierarchical clustering model yielded satisfactory results.

### 4.4 Dbscan

The third unsupervised machine learning method applied was the dbscan model to identify the visit intents on the studied website. Whilst trying to minimize the number of noise points, the four-cluster solution proved to be the most appropriate in the context of this study when employing the dbscan method. Tables 10 and 11 further illustrate the cluster profiles per segment.

Upon profiling the clusters of the dbscan model, cluster one represented visits that imply accidental landing on the web page with very low engagement with the website. Cluster one maintained the highest bounce rate, minimal duration, the furthest geo-proximity with on average two pages viewed per visit. Cluster two represented visits that express main interest in the “contact-us” page alone. Cluster three comprised visits that expressed primary interest in the “courses” and “short courses” page whilst cluster four portrayed primary interest in the “apprenticeships” webpage. Across clusters two, three and four, visits were interested in specific web pages. It was also observed that cluster zero, which the model termed as the noisy data points, represented visits that were relatively the most engrossed, spent the most amount of time on the webpage with a high volume of web pages visited. According to the cluster silhouette coefficients as tabulated in Table 12, the dbscan clustering model resulted in satisfactory results. Clusters one, two and three recorded high silhouette coefficients. Cluster one represented the high-engagement group of visits that naturally expressed a high variance in behaviour patterns (visits spending a longer duration on the website, following unique page paths, and visiting different webpages). Cluster four recorded a poor silhouette score which could be attributed to the cluster size.

## 5 Concluding Remarks

This paper sought to understand the intents behind the website visits of a South African informative website. In doing so, the study employed three unsupervised machine learning models belonging to different clustering families, namely, the k-means, hierarchical and dbscan clustering methods. Across the three different clustering techniques, it was evident that five distinct visit intents were observed in the data. An intent described as “engrossed” was evident in the results. The engrossed segment represented the group of visits that were highly engaged with the website, spent a long duration on the website, visited multiple pages and were often within close proximity to the corporate co-ordinates (Google analytics tracking supplies the co-ordinates of the device whilst on the website). An intent described as “seekers” were discovered. The seekers represented a group of visits that went online, and visited specific webpages, retrieved what was needed and thereafter exited the website. An intent described as “accidental” were discovered. The accidentals were visits that shared a high bounce rate, just stepped into the website then hopped off. It was noticed that the accidentals contained most of the foreign web visitors. An intent that can be described as the “drop-offs” were discovered. The drop-offs would enter the website, explore a few pages, and then afterwards drop off with low to medium engagement on the website. And lastly, an intent that can be described as “get-in-touch” were discovered. The “get-in-touch” represented visits that had primary interest in the “contact-us” web page and thereafter immediately exited the website. Table 13 shares the intents that were discovered by three unsupervised machine learning models.

The k-means method has identified all intents but merged the “accidentals” and “drop-offs” into one cluster. Furthermore, the k-means model, further split the engross segment into a moderate engaging visits and high engaging visits. The dbscan method was also able to identify the common intents but split the seekers into two small volume intents whilst it merged the accidentals and drop-offs. Furthermore, the “engrossed” visits were all labelled as noise data points by the dbscan method. Upon comparison of the subsequent clusters generated by each of the three employed unsupervised machine learning methods, the hierarchical clustering displayed superior results. The hierarchical cluster had successfully isolated the five intents portrayed within the data, the cluster sizes were satisfactory, and the clusters maintained superior homogeneity as quantified by the silhouette coefficients relative to the k-means and dbscan models. Across the three unsupervised machine learning models employed, all models have performed exceptionally well in finding meaningful clusters within the data. However, in the context of this study, the hierarchical model resulted in the results that bested suited the research needs of this study on the observed data.

### 5.1 Future Work

This study was constrained to a single website, that by design was informative with no login facility on a South African engineering and engineering training corporate. The underlying intents that emerged were thus based on the studied website. Future research that could be conducted (using the methodology above) across websites of different industries and geography.

## Figures and Tables

**Table 1:** Research gap

Related Work	Difference
Idowu and Kattukottai [9]	The related work clustered purchase behaviour whilst this paper clustered the underlying visit intents. Whilst purchase clusters are important, an intent model is equally important as not every visitor would commit to a purchase and not all websites have purchase applications
Porsche et al. [10]	The related work employed custom metrics and descriptive statistics to track visitors reading behaviour. However, this paper employs clustering models which is an advanced analytics technique that would allow better generalization of the underlying intents as opposed to descriptive statistics
Domazet and Simovic [11]	The related work employed descriptive statistics to measure a website’s performance. However, this paper employs clustering models which is an advanced analytics technique that would allow better generalization of the underlying intents as opposed to descriptive statistics
Jonathan et al. [12]	Within the related work, the author does not discuss much about the analytics employed. Nonetheless, the audience behaviour expected on a church website would fundamentally differ from the studied website within this paper
Semeradova and Weinlich [13]	The related work here proposed web analytics using the Google Analytics interface which was at an aggregate level. Aggregate analytics will provide a high-level overview which assumes that every visitor has behaved in this manner. The methods employed within this paper allow for better visitor profiling
Kalyankar and Anute [14]	The related work did not employ clustering methods to understand the visitors’ behaviour. However, this paper employs clustering models which is an advanced analytics technique that would allow better generalization of the underlying intents as opposed to descriptive statistics
Cirlugea et al. [15]	The related work focused on the effect of marketing on website volumes. However, did not discuss the quality of the visit by detailing the visitors’ activities once on the website
Rosqa and Ati [16]	The related work here was primarily exploratory
Pirvu and Anghel [17]	The aim of the related work here was to predict behaviour but the authors did not explain the different types of behaviours or events being predicted. A clustering method (as proposed within this paper) should have been first determined
Mariyapillai and Pratheepan [18]	The related work here was primarily exploratory to determine the geo-location of the visits
Stelian and Stoicu-Tivadar [19]	The related work here was primarily exploratory with main interest in visitor interaction with virtual bone structures. Clustering may not be appropriate within this application if a visitor was guided through the application as opposed to a website where a visitor is free to behave as desired

**Table 2:** Notations used within the study

Notation	Description
*k*	A real valued integer to represent the number of clusters
*x*	Represents a single data point of feature *X*
ϵ	A predefined hyper-parameter that sets the distance between a point and its neighbours
*n*	Minimum number of points within cluster

**Table 3:** Features explored within unsupervised machine learning

Feature name	Data type	Feature description
Accreditations	Numeric	Count of visits the user made to this page within each session
Apprenticeship	Numeric	Count of visits the user made to this page within each session
Bounces	Binary	Flags if the session was a single page visit only
Contact-us	Numeric	Count of visits the user made to this page within each session
Courses	Numeric	Count of visits the user made to this page within each session
Customised-engineering-trading	Numeric	Count of visits the user made to this page within each session
daysSinceLastSession	Numeric	The number of days a user is returning to the website
Distance	Numeric	The Euclidean distance between the user’s co-ordinates and the company’s co-ordinates (owner of the website)
Engineering-academic-studies	Numeric	Count of visits the user made to this page within each session
Engineering-Trade	Numeric	Count of visits the user made to this page within each session
Hits	Numeric	Represents any action on a webpage that results in data being sent to Google Analytics (such as page clicks, etc.)
Home	Numeric	Count of visits the user made to this page within each session
OrganicSearches	Binary	Flag to indicate if the user organically constructed a search that resulted in landing onto the webpage (a web link was not clicked)
Pageviews	Numeric	The number of instances a page was loaded (or reloaded)
sessionCount	Numeric	An indicator of the nth time the user has accessed the website
SessionDuration	Numeric	The duration of the session (seconds)
Short-courses-skilled-programmes	Numeric	Count of visits the user made to this page within each session
Trade-test-arpl	Numeric	Count of visits the user made to this page within each session
University-of-technology-uot	Numeric	Count of visits the user made to this page within each session

**Table 4:** K-means cluster profiling across web metrics

K-means clusters	Cluster size	% Size	Avg SessionDuration	Avg bounces rate (%)	Avg organic search rate (%)	*Avghits*	Avg sessionCount	Avg daysSinceLastSession	Avg distance	Avg pageviews
1	988	15.0	159.84	7.0	55.0	5.42	1.55	3.42	16.14	5.40
2	3260	49.6	43.98	34.0	40.0	2.47	1.54	2.48	32.51	2.45
3	1066	16.2	398.56	0.0	66.0	11.11	1.39	3.18	7.17	11.02
4	778	11.8	196.44	7.0	61.0	8.41	1.38	2.97	7.35	8.36
5	477	7.3	611.91	0.0	70.0	26.02	1.27	3.00	4.74	25.94

**Table 5:** K-means cluster profiling across web page visits

K-means clusters	Home page index	Accreditations page index	Apprenticeship page index	Contact-us page index	Courses page index	Engineering-trade page index	Engineering-academic-studies page index	Customised-engineering-trading page index	Short-courses-skilled-programmes page index	Trade-test-arpl page index	University-of-technology-uot page index
1	0.94	0.09	0.08	1.15	0.26	0.02	0.06	0.02	0.00	0.11	0.04
2	0.86	0.05	0.05	0.00	0.24	0.02	0.04	0.01	0.00	0.05	0.03
3	1.52	0.22	0.66	0.11	1.98	0.37	0.11	0.20	0.00	0.66	0.26
4	1.15	0.11	0.11	0.15	0.97	0.16	0.17	0.09	1.21	0.20	0.10
5	1.95	0.64	0.79	0.45	2.10	0.82	1.07	0.92	1.18	1.17	0.82

**Table 6:** K-means cluster summary

K-means clusters	Silhouette coefficient	Outstanding attributes
1	0.31	Over-index on “contact-us” page, low bounce rate, moderate visit duration
2	0.40	Very low session duration, very low page views, high bounce rate, furthest distance from the corporate location
3	−0.05	Fairly high session duration, fairly high pageviews, low bounce rate
4	0.13	Over-index on “courses page” and “short-courses page”, moderate session duration, low bounce rate
5	0.01	Very high session duration, very high page views, very close geo-proximity to the corporate coordinates, low bounce rate and high organic search rate

**Table 7:** Hierarchical cluster profiling across web metrics

Hierarchical clusters	Cluster size	% Size	Avg SessionDuration	Avg bounces rate (%)	Avg organic search rate (%)	Avg hits	Avg sessionCount	Avg daysSinceLastSession	Avg distance	Avg Pageviews
1	2509	38.2	306.63	6.0	57.0	11.67	1.63	6.02	8.26	11.60
2	551	8.4	202.87	9.0	62.0	7.96	1.35	1.24	8.44	7.88
3	720	11.0	4.06	56.0	22.0	1.54	1.03	0.02	124.30	1.54
4	1994	30.4	91.47	29.0	51.0	2.92	1.48	0.15	4.30	2.90
5	795	12.1	131.41	9.0	53.0	4.53	1.50	3.14	17.77	4.52

**Table 8:** Hierarchical cluster profiling across web page visits

Hierarchical clusters	Home page index	Accreditations page index	Apprenticeship page index	Contact-us page index	Courses page index	Engineering-trade page index	Engineering-academic-studies page index	Customised-engineering-trading page index	Short-courses-skilled-programmes page index	Trade-test-arpl page index	University-of-technology-uot page index
1	1.28	0.35	0.53	0.22	1.30	0.38	0.33	0.30	0.34	0.49	0.35
2	1.15	0.00	0.14	0.20	0.96	0.03	0.21	0.05	1.19	0.18	0.02
3	0.68	0.00	0.00	0.00	0.03	0.00	0.00	0.00	0.00	0.01	0.00
4	1.07	0.00	0.00	0.00	0.46	0.00	0.00	0.00	0.00	0.14	0.00
5	0.89	0.00	0.00	1.15	0.21	0.00	0.00	0.00	0.00	0.09	0.00

**Table 9:** Hierarchical cluster summary

Hierarchical clusters	Silhouette coefficient	Outstanding attributes
1	−0.21	High session duration, high pageviews, low bounce rate
2	0.15	Over-index on “short-courses” page and “courses” page, moderate session duration, low bounce rate, highest organic search rate
3	0.60	Very low session duration, very low page views, high bounce rate, furthest distance from the corporate location
4	0.38	Moderate session duration, brief page views, moderate bounce rate, close proximity to corporate co-ordinates
5	0.39	Over-index on “contact-us” page, low bounce rate, moderate session duration

**Table 10:** Dbscan cluster profiling across web metrics

Dbscan clusters	Cluster Size	% Size	Avg SessionDuration	Avg bounces rate (%)	Avg organic search rate (%)	Avg hits	Avg sessionCount	Avg daysSinceLastSession	Avg distance	Avg pageviews
0	3151	48.0	331.19	5.0	56.0	11.17	1.62	5.74	10.68	11.10
1	2440	37.1	26.41	40.0	42.0	2.05	1.37	0.16	39.13	2.04
2	592	9.0	58.72	8.0	58.0	3.78	1.34	0.15	11.27	3.77
3	270	4.1	78.29	15.0	60.0	5.07	1.21	0.11	5.33	5.05
4	116	1.8	60.01	3.0	67.0	3.44	1.30	0.13	2.87	3.38

**Table 11:** Dbscan cluster profiling across web page visits

Dbscan clusters	Home page index	Accreditations page index	Apprenticeship page index	Contact-us page index	Courses page index	Engineering-Trade page index	Engineering-academic-studies page index	Customised-engineering-trading page index	Short-courses-skilled-programmes page index	Trade-test-arpl page index	University-of-technology-uot page index
0	1.32	0.28	0.40	0.30	1.28	0.31	0.30	0.25	0.39	0.54	0.28
1	0.91	0.00	0.00	0.00	0.24	0.00	0.00	0.00	0.00	0.00	0.00
2	0.78	0.00	0.00	1.08	0.06	0.00	0.00	0.00	0.00	0.00	0.00
3	0.95	0.00	0.00	0.00	0.80	0.00	0.00	0.00	1.09	0.00	0.00
4	0.46	0.00	1.17	0.00	0.16	0.00	0.00	0.00	0.00	0.00	0.00

**Table 12:** Dbscan cluster summary

Dbscan clusters	Silhouette coefficient	Outstanding attributes
0	−0.11	High visit duration, high pageviews, low bounce rate
1	0.45	Very low session duration, very low page views, high bounce rate, furthest average distance from the corporate location
2	0.45	Over-index on “contact-us” page, low bounce rate, moderate session duration
3	0.21	Over-index on “short course” page, high index on the “courses” page, moderate session duration, low bounce rate
4	−0.34	Over-index on “apprenticeship” page, moderate session duration, low bounce rate, high organic search rate

**Table 13:** Web visit cluster comparison

Method	Cluster	Size (%)	Silhouette coeff.	Persona
K-means	1	15.04	0.31	Get-in-touch
	2	49.63	0.40	Accidentals/Drop-offs
	3	16.23	−0.05	Engrossed: Moderate engagement
	4	11.84	0.13	Seekers
	5	7.26	0.01	Engrossed: High engagement
Hierarchical	1	38.19	−0.21	Engrossed
	2	8.39	0.15	Seekers
	3	10.96	0.60	Accidentals
	4	30.35	0.38	Drop-offs
	5	12.10	0.39	Get-in-touch
DBSCAN	0	47.97	−0.11	Noise (resembles Engrossed)
	1	37.14	0.45	Accidentals/Drop-offs
	2	9.01	0.45	Get-in-touch
	3	4.11	0.21	Seekers: Short-courses
	4	1.77	−0.34	Seekers: Apprenticeships

## Data Availability

None. Unfortunately, the data belongs to the owner of the website. The study was completed for a South African company who hold rights to the data. The author has promised security of the data and ensured that the results have be shared in a compliant manner.

## Code availability

R code available upon request.
